# Cancer trials in sub-Saharan Africa: Aligning research and care

**DOI:** 10.1371/journal.pmed.1002351

**Published:** 2017-07-10

**Authors:** Satish Gopal

**Affiliations:** 1 Malawi Cancer Consortium, Lilongwe, Malawi; 2 UNC Project-Malawi, Lilongwe, Malawi; 3 Lineberger Comprehensive Cancer Center, University of North Carolina at Chapel Hill, Chapel Hill, North Carolina, United States of America; 4 Institute for Global Health and Infectious Diseases, University of North Carolina at Chapel Hill, Chapel Hill, North Carolina, United States of America; 5 Gillings School of Global Public Health, University of North Carolina at Chapel Hill, Chapel Hill, North Carolina, United States of America; 6 University of Malawi College of Medicine, Blantyre, Malawi

## Abstract

Satish Gopal discusses the challenges of deliverable cancer care and cancer trials in sub-Saharan Africa as well as a potential framework for overcoming these challenges.

**Summary points**

- There is an extreme scarcity of evidence to guide cancer treatment in sub-Saharan Africa (SSA), as well as major differences between SSA and resource-rich settings regarding cancer treatment infrastructure.
- A possible framework for conceptualizing cancer clinical trials in SSA is proposed, and key issues related to equipoise, innovation, and efficiency are addressed within the SSA context.
- Strongly aligned cancer care and research agendas can generate forward progress for cancer treatment in the region and globally impactful clinical science that can change treatment paradigms even in resource-rich settings.

## Introduction

Cancer burden is increasing in sub-Saharan Africa (SSA), with more than 600,000 estimated new cancer cases in 2012 and age-standardized incidence increases of 10%–20% in most countries between 2005 and 2015 [1,2]. Major regional limitations in pathology, surgery, medical oncology, radiation, and palliation have been extensively described and contribute directly to worse cancer outcomes in SSA than in high-income countries [3]. However, even when treatment is available, there is a remarkable and unacceptable scarcity of high-grade evidence to guide the application of cancer treatment for the 1 billion people living in SSA (Table 1). Interest, investment, and infrastructure are gathering to address this problem. Two cooperative groups sponsored by the United States National Cancer Institute (NCI) have initiated activities in the region, the AIDS Malignancy Consortium (AMC) for human immunodeficiency virus (HIV)-associated malignancies and a second network for pediatric Burkitt lymphoma. Other similar efforts are ongoing. However, conceptualizing cancer treatment trials that are sufficiently informative and appropriate to pursue in SSA continues to prove challenging, even for experienced investigators in the region. This Essay, based on experience in Malawi and broader participation in regional clinical trial groups, elucidates some of these challenges and possible ways forward.

**Table 1:** Key completed and ongoing clinical trials evaluating specific cancer treatment interventions among adults in sub-Saharan Africa.

Disease area	Countries	Years	Patients	Intervention	Main results
Study					
**Non-Hodgkin lymphoma**					
Mwanda et al. [4]	Kenya and Uganda	2001–2005	49 HIV+	Dose-modified oral chemotherapy	Median EFS 7.9 months, median OS 12.3 months, 33% 5-year OS
NCT02660710	Malawi	2016–	20 HIV+, 20 HIV-	Rituximab + CHOP	Pending
NCT01775475	Kenya, Malawi, Uganda, and Zimbabwe	2016–	90 HIV+	CHOP versus dose-modified oral chemotherapy	Pending
**Kaposi sarcoma**					
Olweny et al. [5]	Zimbabwe	1994–1999	495 HIV+	Supportive care versus radiotherapy versus oral etoposide versus DVB	Oral etoposide improved QOL more than the other 3 arms
Mosam et al. [6]	South Africa	2003–2009	112 HIV+	ART versus ART + chemotherapy	ART + chemotherapy improved KS response at 12 months (66% versus 39%)
Martin et al. [7]	Uganda	2007–2012	224 HIV+	ART with PI versus ART with NNRTI	No difference between the arms in indication for chemotherapy or death
NCT01435018	Malawi, Kenya, South Africa, Uganda, and Zimbabwe	2013–	706 HIV+	BV versus paclitaxel versus oral etoposide (advanced disease)	Pending
NCT01352117	Malawi, Kenya, South Africa, Uganda, and Zimbabwe	2011–2016	192 HIV+	ART versus ART + oral etoposide (limited disease)	Pending
**Cervical cancer**					
NCT01590017	Zimbabwe	2014–	41 HIV+	Cisplatin + radiotherapy	Pending

Published studies and those registered at ClinicalTrials.gov were included. The table excludes intervention studies aimed at preventing rather than treating cancer and also intervention studies that exclusively enrolled patients in South Africa as part of multinational trials designed for high-income settings. ART = antiretroviral therapy; BV = bleomycin and vincristine; CHOP = cyclophosphamide, doxorubicin, vincristine, and prednisone; dose-modified oral chemotherapy = lomustine, etoposide, cyclophosphamide, and procarbazine; DVB = dactinomycin, vincristine, and bleomycin; EFS = event-free survival; HIV = human immunodeficiency virus; NNRTI = non-nucleoside reverse transcriptase inhibitor; OS = overall survival, PI = protease inhibitor; KS = Kaposi sarcoma; QOL = quality of life.

### What is equipoise for cancer trials in SSA?

One difficulty is deciding which established treatments in resource-rich settings can be straightforwardly generalized to SSA and which require specific demonstrations of safety and efficacy in SSA. There is no established framework for such decisions, and there are significant differences of opinion regarding this issue. Uncertainty results from scarce regional data and important SSA differences from resource-rich settings where cancer treatments have been studied. These differences include host genetics and metabolism; tumor biology; endemic burden of infectious pathogens, including HIV; and profound differences in general healthcare infrastructure, any of which could dramatically alter how specific treatments perform relative to high-income countries.

As a result, one might argue that SSA is sufficiently different from resource-rich settings where existing evidence for cancer treatment has been generated and that all studies should be redone in the region. Indeed, for an arguably much less intensive intervention, antiretroviral therapy (ART), the HIV community first undertook proof-of-principle studies as a foundation for larger scale-up, to mitigate concerns that poor adherence and rampant HIV resistance would result without intensive monitoring [8,9]. Additionally, even in high-income countries, real-world effectiveness is often very different from within-trial efficacy [10]. Thus, extrapolating not only from trials to routine care settings but from resource-rich settings to SSA may be too much to ask of SSA ministries of health and medicine licensing boards without first building additional evidence, as these agencies are tasked with applying scarce resources to optimally benefit public health. Alternatively, some experts argue that no further evidence is needed to apply international standards of care for cancer patients in SSA. This is admirable, but in countries like Malawi, such dogmatic “universality” arguments are unrealistic and possibly even harmful—for example, if high-intensity cancer treatments from high-income countries are applied without suitable levels of support. Despite billions of dollars in donor assistance over many decades, the fact remains that the Malawi public sector does not have remotely comparable capacity to the United States for delivering high-intensity cancer treatment.

Therefore, a nuanced middle approach is likely most sensible, with clearer articulation of evidentiary standards for including antineoplastic essential medicines in World Health Organization (WHO) lists [11]. For instance, the uniquely favorable therapeutic ratio for imatinib in chronic myelogenous leukemia (CML) led to the Novartis Glivec International Patient Assistance Program (GIPAP), perhaps the most far-reaching global oncology pharmaceutical access program ever attempted, without first redemonstrating efficacy in low-income countries benefitting from GIPAP [12]. Similar arguments could be made for trastuzumab for HER2-positive breast cancer, for which worldwide access remains limited, and possibly rituximab for CD20-positive non-Hodgkin lymphoma (NHL). Both medicines are notable for having available biosimilars with published evidence for equivalence [13,14], although even these biosimilars remain economically out of reach for many SSA countries. For rituximab, we have argued in Malawi that high opportunistic infection burden, high HIV prevalence among adult NHL patients, and limited infusion and supportive care including absent hematopoietic growth factors warrant rituximab introduction within defined patient populations in the context of a prospective clinical trial [15]. For our study, we are using the commercially available biosimilar, with research ethics committee approval and data safety monitoring board oversight to ensure patients are not harmed by excessive unanticipated hypersensitivity, neutropenia, lymphodepletion, or infectious complications, before advocating more widespread application. Developing a robust regional framework to prioritize cancer interventions requiring further study, through consensus generated in SSA, would be valuable.

### What is innovation in SSA, and can SSA cancer trials have global importance?

Given uncertainties about clinical equipoise in SSA, a related question is what constitutes cancer innovation in SSA, as well as whether SSA cancer trials can have global rather than regional importance. Indeed, the global cancer research community was remarkably fortunate with its first SSA activities focused on endemic Burkitt lymphoma, which led to the discovery of Epstein-Barr virus (EBV), the *MYC* oncogene, and modern principles of combination chemotherapy [16]. Similar discoveries from contemporary efforts might not be so readily forthcoming. Indeed, given implicit justifications for many cancer studies in the region, it may be worth stating that SSA cancer patients do not exist primarily to discover new viruses or gene mutations and that the overarching goal of trials must be to generate knowledge that extends life and reduces morbidity for cancer patients in SSA. Especially when modern sequencing and bioinformatics are increasingly available in more economically advanced countries, like China, India, or Brazil, where cancer pathogenesis may at least partially resemble SSA, there may be limited future opportunities in SSA to discover entirely new fundamental biologic insights for cancer. This is particularly true when many experiments require shipping tissues to high-income countries, raising important and complex ethical, regulatory, and cultural considerations that must first be addressed. Indeed, we have undertaken among the first molecular profiling studies in SSA for 3 high-burden cancers in Malawi (Kaposi sarcoma, esophageal squamous cell carcinoma, and diffuse large B-cell lymphoma), which have often reproduced findings from other parts of the world, though in our view this makes them no less important for SSA [17–19].

Moreover, in pursuit of innovation, the willingness to invest in incremental advances for cancer patients in high-income countries has often not translated to SSA. New “blockbuster” cancer therapies, like brentuximab vedotin for Hodgkin lymphoma or immune checkpoint inhibitors for many cancer types, are appropriately heralded with incremental efforts to define their utility across a range of clinical niches in high-income countries. In SSA, among all cancers, pediatric Burkitt lymphoma has benefitted from the strongest commitment to achieving rigorous incremental advances under local conditions, as in other international pediatric oncology groups [20–24]. Nevertheless, the appropriate first-line chemotherapy regimen for this disease in SSA remains uncertain half a century after its discovery in Uganda, with substantial regional variation in treatment approaches and reported outcomes despite often quite similar patients and settings.

Whatever the difficulties encountered in SSA, several lines of inquiry could lead to globally impactful and innovative clinical science. First, certain cancers occur with unique frequency in SSA and can only be studied in large numbers if patients are included from this region. Examples include endemic Burkitt lymphoma, Kaposi sarcoma, and conjunctival squamous cell carcinoma. Second, with the availability of highly effective noncytotoxic treatments for cancer and the identification of patient subgroups with particularly favorable prognoses like head and neck squamous cell carcinoma caused by human papillomavirus (HPV), there is increasing interest in treatment de-escalation in high-income countries. De-escalated treatment, however, may be difficult to accept in resource-rich settings, where there is a natural reluctance to deviate substantially from aggressive treatment protocols proven to result in excellent outcomes and where undertreatment is typically viewed as a graver error than overtreatment. Given real-world barriers to universally applying regimens from high-income countries as articulated above, de-escalated strategies may be highly appropriate and even necessary in SSA, presenting opportunities for “leapfrog” advances by evaluating regimens that could be applicable in high-income countries. Finally, development of noninvasive biomarkers for cancer diagnosis, prognosis, and response assessment would be particularly valuable in SSA where pathology and diagnostic imaging are limited, especially when these have realistic potential for implementation in SSA. As an example, we have pursued evaluation of quantitative plasma EBV DNA in endemic Burkitt lymphoma, using instruments already in place for HIV RNA monitoring, to guide risk-adapted and response-guided therapy analogous to fluorodeoxyglucose positron emission tomography (FDG-PET) in high-income countries [25–27].

### How can clinical trial efficiency be optimized in SSA?

In addition to biologic heterogeneity, cancer trials in SSA will have to cope with marked heterogeneity in populations and health infrastructure across settings. Moreover, health infrastructure in SSA is changing constantly and at times quite rapidly. Trials predicated on absent radiotherapy or hematopoietic growth factors, for instance, might be undermined by the sudden introduction of these adjunctive treatments within relatively short periods and with this introduction typically not achieved in a uniform manner. Given these complicating factors, plus the enormous evidence deficit for cancer treatment in SSA, as well as the enormous need to build trust and prove to SSA stakeholders that cancer research unequivocally benefits public health, there is no region of the world in greater need of flexible, adaptive trial designs that appropriately respond to accumulating evidence or changing conditions on the ground, ensure maximum therapeutic benefit for trial participants, and minimize financial and time expenditures. Again, this is an opportunity for true leapfrog advances within nascent cancer trial networks in the region, which do not have to be unduly constrained by traditional protocol development approaches that may be too rigid and slow to drive oncology care forward in SSA.

### A way forward

Taking these issues into consideration, a possible framework for conceptualizing cancer clinical trials in SSA is proposed in Table 2. This scheme is not intended to address more fundamental aspects of clinical trial design, larger economic considerations related to global cancer care, or complex ethical issues in global health, but rather, it is meant to highlight key attributes for an otherwise well-designed study that might make it particularly apt for SSA. There is admittedly tremendous subjectivity in judging a given study according to these traits, and ideally, such judgments would reside within groups of cancer investigators in SSA rather than meetings outside SSA, where a deficit of high-grade evidence from the region makes it easy to distort what may or may not be appropriate.

**Table 2:** Ideal attributes of a cancer treatment trial in sub-Saharan Africa.

The trial addresses local disease burden in an inclusive rather than exclusive manner.
• The cancer is seen frequently in the region.
• The trial includes typical patients with this cancer in the region (e.g., studies that include advanced stage disease will generally be more valuable for most cancer types).
• The trial includes patients from urban and rural settings and from different tribal/ethnic groups.
The trial makes a resource-appropriate standard of care available for all participants.
• This standard may not be exactly the same as that of high-income countries but, likewise, should not simply be what is usually available for routine care if this is not an acceptable standard.
The trial provides new data for an intervention for which there is not sufficient published experience in the region.
The intervention might be applied to patients in high-income countries if successful, although this is not essential to justify the trial.
• Where these exist, opportunities to pursue a new global standard are important, rather than recurrently describing suboptimal or modestly effective treatment in challenging settings.
The trial is designed and analyzed in a manner that maximizes time and cost efficiency.
The anticipated cost/benefit ratio of the intervention is comparable to other established health interventions in the region.
When included, clear hypotheses underlie correlative studies using biologic specimens sent outside the region, to justify the effort and expense needed to collect and ship these materials.
Subsequent trials or implementation steps appropriate for the region can be clearly articulated before the study is initiated, for both positive and negative outcomes.
• There is commitment to pursue subsequent trials or implementation steps once the study is completed, informed by its results.
The trial’s appropriateness and value are affirmed by local community representatives or patient groups prior to implementation.

## Conclusions

There is a growing recognition in high-income countries that care and research agendas for cancer must be brought closer together, for clinical, scientific, and economic reasons. In SSA, there are simply not sufficient resources to allow these agendas to diverge and/or compete, a luxury born of having excess resources in the first place. Care and research must be aligned and even considered the same—for example, as in international pediatric oncology groups, in which there has been a decades-long commitment to treating cancer on harmonized protocols across centers, to drive clinical science forward for specific diseases and individual children [28]. A potential framework for achieving similar progress in SSA is proposed, to avoid excessive external investment in studies that do not substantially inform or improve care in SSA. With these challenges at the forefront, continued regional efforts and momentum to generate forward progress are eagerly anticipated, by cancer policy makers, clinicians, and patients above all.

## Abbreviations

AMC: AIDS Malignancy Consortium
ART: antiretroviral therapy
BV: bleomycin and vincristine
CHOP: cyclophosphamide, doxorubicin, vincristine, and prednisone
CML: chronic myelogenous leukemia
DVB: dactinomycin, vincristine, and bleomycin
EBV: Epstein-Barr virus
EFS: event-free survival
FDG-PET: fluorodeoxyglucose positron emission tomography
GIPAP: Glivec International Patient Assistance Program
HIV: human immunodeficiency virus
HPV: human papillomavirus
KS: Kaposi sarcoma
NCI: National Cancer Institute
NHL: non-Hodgkin lymphoma
NNRTI: non-nucleoside reverse transcriptase inhibitor
PI: protease inhibitor
QOL: quality of life
SSA: sub-Saharan Africa
WHO: World Health Organization
